# T cell subsets in cervical cancer tumor microenvironment: advances and therapeutic opportunities

**DOI:** 10.3389/fimmu.2025.1612032

**Published:** 2025-06-05

**Authors:** Weilin Guo, Lan Dai, Lihua Qiu

**Affiliations:** ^1^ Department of Obstetrics and Gynecology, Ren Ji Hospital, Shanghai Jiao Tong University School of Medicine, Shanghai, China; ^2^ Shanghai Key Laboratory of Gynecologic Oncology, Ren Ji Hospital, Shanghai Jiao Tong University School of Medicine, Shanghai, China

**Keywords:** T cell, tumor microenvironment (TME), cervical cancer, immune regulation, immunotherapy

## Abstract

Cervical cancer is the third most common malignancy among Chinese women in both incidence and mortality. Its progression is closely linked to complex interactions among immune cells within the tumor microenvironment (TME). As key components of the immune landscape, different T cell subsets play diverse and dynamic roles in shaping tumor immunity. This review provides a comprehensive overview of the roles of various T cell subsets in the TME of cervical cancer, with a focus on their distribution, functional heterogeneity, dynamic balance, and variations across different pathological subtypes and disease stages. We also highlight the intricate crosstalk between T cells and other immune cells in the TME and discuss recent advances in T cell-related immunotherapies for cervical cancer, including immune checkpoint inhibitors and HPV-targeted vaccines. By elucidating the roles of distinct T cell subsets and relevant immunotherapeutic approaches within the TME, this review provides insights into potential therapeutic targets and approaches for improving cervical cancer treatment and patient outcome.

## 1 Introduction

Cervical cancer is the fourth most commonly diagnosed cancer among women worldwide and ranks third in China. In 2020, China reported 109,741 new cases and 59,060 deaths from cervical cancer. Notably, it tends affect younger women compare to other gynecological cancers. Squamous cell carcinoma (70%) and adenocarcinoma (25%) are the most common histological subtypes (1). The precursors of cervical cancer are categorized as cervical intraepithelial neoplasia grades 1, 2, and 3 (CIN1, CIN2, and CIN3), based on the severity of dysplasia. Persistent human papillomavirus (HPV) infection is a major etiological factor in cervical cancer development (2) and high-risk HPV infections are strongly associated with high-grade CIN (3).

T cells are a critical cellular component of the TME and play a central regulatory role in tumor initiation and progression. The TME refers to the localized, steady-state environment intimately associated with tumorigenesis (4). It includes tumor cells, immune cells, blood vessels, lymphatic vessels, and the extracellular matrix (5). T lymphocytes, as the primary effector cells in cellular immunity, produce cytokines during immune responses to mediate inflammation and modulate the activity of other immune cells (6). They can be classified into various subtypes based on different criteria. Functionally, they can be categorized into cytotoxic T cells (Tc), helper T cells (Th), regulatory T cells (Treg), and memory T cells. According to surface markers, they are divided into αβT cells (65–75%) and γδT cells (1–5%). αβT cells undergo positive and negative selection to differentiate into mature CD4^+^ and CD8^+^ T cells. The degree of CD4^+^ and CD8^+^ T cell infiltration has been shown to correlate with the severity of cervical cancer lesions (7). It has been demonstrated that most solid tumors, including cervical cancer, possess an immunosuppressive TME, which is closely associated with tumor progression (8). The TME of cervical cancer can be categorized into two distinct subtypes: the active immune subtype and the exhausted immune subtype. The active immune subtype is characterized by an enhanced interferon (IFN) signature, increased infiltration of immune cells, including CD8^+^ T cells, macrophages, and dendritic cells, and a robust antitumor response. Conversely, the exhausted immune subtype exhibits immunosuppressive and pro-tumor features, such as stromal activation, wound-healing gene expression, activation of the TGF-β signaling pathway, and diminished CD8^+^ T cell infiltration. Patients who tested negative for the high-risk HPV16/18 strains were more frequently classified under the exhausted immune subtype (9).

T cells are widely recognized as key therapeutic targets in cancer immunotherapy. Current standard treatments for cervical cancer include hysterectomy, radiotherapy, chemotherapy, and chemoradiotherapy (10). However, curative potential remains limited, positioning immunotherapy as a promising alternative. Understanding the alterations in T cell-mediated immunity within the TME of cervical cancer holds promise for advancing its diagnosis and treatment.

This review examines the relationship between various T cell subsets and cervical cancer, with a focus on their distribution, functional heterogeneity, and variations of these subsets across different pathological types and disease stages. Additionally, we discuss the complex interactions between T cells and other immune cells in the TME, as well as recent advances in T cell-related immunotherapies for cervical cancer.

## 2 Different subsets of T cells in the TME of cervical cancer

The functions of T cells in the TME of cervical cancer vary significantly across subsets. Based on T cell receptor (TCR) types, T cells are classified into αβT cells and γδT cells. αβT cells constitute the predominant subset of T cells and undergo both positive and negative selection processes, ultimately differentiating into mature CD4^+^ and CD8^+^ T cells. γδT cells consist of only 1–5% of peripheral blood T lymphocytes. Since they can directly recognize tumor-associated antigens without Major Histocompatibility Complex (MHC) restriction, this attribute makes them one of the most promising candidates for immunotherapy (11). Understanding the distinct roles and functional diversity of these T cell subsets is essential for further elucidating the immune landscape of the cervical cancer TME.

### 2.1 CD8^+^ T cells

CD8^+^ T cells exhibit potent antitumor activity in cancer, and their infiltration level is a commonly used prognostic indicator for cervical cancer. However, increasing evidence suggests that in the TME of cervical cancer, most CD8^+^ T cells are exit in an exhausted state. Although they remain responsive to neoantigens and exhibit marked clonal expansion, they fail to exert effective antitumor functions (12). In cervical cancer tissues, multiple immunosuppressive and immune checkpoint genes are highly expressed (13, 24); these factors inhibit CD8^+^ T cell activity, promoting immune evasion in the TME. A recent study has shown that miR-142-5p secreted by cervical cancer cells can induce IDO expression, directly contributing to CD8^+^ T cell exhaustion and impairing their antitumor functionality in the TME of cervical squamous cell carcinoma (CSCC) (14). Importantly, not all CD8^+^ T cells remain exhausted, as studies have observed expression of immune activation-related genes, suggesting that they retain antitumor capabilities (15).

In cervical cancer, several factors have been identified as influencing prognosis by modulating CD8^+^ T cell levels and activity. IL-33 is an epithelial-derived cytokine that can function as an alarmin within the TME. Zhang et al. demonstrated through semi-quantitative immunohistochemical analysis that elevated IL-33 expression in cervical cancer cells was significantly associated with increased CD8^+^ T cell infiltration and improved prognosis (16). In contrast, a recent study has demonstrated that the IL-33-mediated IL-33-ILC2s-IL-13-M-MDSCs axis plays a primary tumor-promoting role in cervical cancer. Its excessive activation significantly accelerates cervical cancer progression (17). Although primarily based on animal models, this study also observed consistent trends in human samples, supporting the clinical relevance of their findings. These studies differ substantially in terms of experimental models, detection methods, and the immune pathways investigated. The contradictory findings highlight the dual role of IL-33 in tumor immunity. Its function may be shaped by various factors, including regulatory signaling pathways, receptor distribution, cellular localization, and the immune status of the tumor. Further mechanistic studies across different pathological stages and immune contexts are warranted to clarify its immunoregulatory functions. GLUT1 expression has been identified as an independent prognostic factor in cervical cancer patients. Studies have shown that high GLUT1 expression impairs the activity of CD8^+^ T cells and Th1 cells, altering the tumor immune microenvironment in HPV16-positive cervical cancer patients. The resulting immune evasion may contribute to treatment failure (18). Additionally, low basic leucine zipper ATF-like transcription factor 2 (BATF2) expression *via* its interaction with c-JUN (19), significantly promotes cervical cancer progression. This is accompanied by reduced CD8^+^ T cell infiltration and a more exhausted phenotype, suggesting that BATF2 deficiency impairs the antitumor immune response (20). T cell-intrinsic stimulator of interferon genes (STING) expression serves as an independent predictor of poor prognosis in HPV-positive cervical cancer patients, with STING levels negatively correlated with CD8^+^ T cell density (21). In contrast, activation by the STING agonist MSA-2, significantly increased CD8^+^ T cell quantity and activity in the TME, as well as levels of immune cells such as NK cells and M1 macrophages (22). These findings highlight the distinct cell-type-specific effects of STING signaling: T cell intrinsic STING activation may suppress T cell function, whereas systemic administration of STING agonists may enhance the overall antitumor immune response by improving the TME and promoting immune cell interactions. This distinction suggests that, when designing STING-targeted therapeutic strategies, both the cellular background and mode of activation should be carefully considered to avoid aberrant STING activation in T cells, which may lead to functional exhaustion and ultimately counteract the therapeutic efficacy. Tscm (Antigen-specific stem-like memory T cells) represent a novel memory T cell subset characterized by strong stem-like properties, Zhang et al. confirmed that high levels of CD8^+^ Tscm may help prevent HPV infection from progressing to cancer (23). Additionally, **Table 1** provides a summary of the factors related to exhausted state of CD8^+^ T cells in cervical cancer mentioned in the text.

**Table 1:** Summary of factors related to exhausted state of CD8^+^ T cells in cervical cancer.

Category	Specific Factor	Mechanism or Observation
Exosomes	miR-142-5p	miR-142-5p induces IDO expression, directly contributing to CD8^+^ T cell exhaustion (14)
Transcription factor	BATF2	Cell with low BATF2 expression exhibits a more exhausted phenotype and reduced IFN-γ production (20)
Immune checkpoint molecules	PDCD1, LAG3, HAVCR2,	These immune checkpoint molecules are found highly expressed in exhausted CD8^+^ T cells through scRNA-Seq analysis (24)
Gene	*GZMK, CXCL13*	Immune exhausted and neoantigen non-reactive CD8^+^ T cells show low expression of *CXCL13* and high expression of *GZMK (* 15)
	*CXCL13, ENTPD1, TOX, PDCD1, TIGIT, HAVCR2, LAG3, KIR2DL4*	Immune exhausted and neoantigen reactive CD8^+^ T cells highly express these genes (12, 15)

Future studies should explore the key pathways regulating CD8^+^ T cell exhaustion and functional reprogramming to achieve precise and durable activation of their antitumor functions, thereby enhancing immunotherapeutic efficacy and improving patient outcomes.

### 2.2 CD4^+^ T cells

Within the TME, CD4^+^ T cells exhibit dual functions: some differentiate into Th, which assist other immune cells in mounting antitumor responses, while others become Tregs, which suppress excessive immune activity to preserve immune homeostasis (15). Both Th cells and Treg cells originate from naïve T cells upon antigen stimulation (6).

#### 2.2.1 Th cells

Th cells can be classified into Th1, Th2, and Th17 subtypes, with Th17 cells receiving extensive attention in cervical cancer research. Th17 cells are a subset of CD4^+^/IL-17^+^ T helper cells that exhibit both antitumor and pro-tumorigenic properties. On one hand, they can indirectly mediate antitumor effect by recruiting other effector immune cells, such as NK cells and tumor-infiltrating IFN-γ effector T cells (25), as well as by activating CD8^+^ T cells (26). On the other hand, Th17 cells can be recruited to the tumor site by stromal or tumor cells through the secretion of chemokines such as IL-12 and IL-23 (27), where they promote tumor progression by inducing angiogenesis and immune suppression.

Most evidence suggest that Th17 cells primarily exert pro-tumorigenic effects: their infiltration in tumor tissues correlates with poor patient prognosis and increases progressively with disease progression (28). Walch-Ruck Heim et al. further demonstrated that C/EBPβ signaling pathway is activated in stromal fibroblasts of cervical cancer *via* IL-6 secretion, thereby inducing CCL20 expression and recruiting Th17 cells infiltration (29). In addition, elevated Th17 cell levels in peripheral blood are closely associated with advanced clinical stages of cervical cancer and the digress of lymph node metastasis (30). As the main cytokine secreted by Th17 cells, IL-17 has received considerable attention in the related studies. In early-stage cervical cancer, high IL-17 expression was identified as an independent prognostic factor for poor outcomes (29), as IL-17 promotes cervical cancer cell proliferation and enhances intercellular adhesion. Such observation suggests pro-tumorigenic role of IL-17 during the early stages of tumorigenesis. This is consistent with the findings by Alves et al. showed that Th17 cells activate chronic inflammatory responses through the secretion of inflammatory factors, such as IL-17, thereby promoting the expansion of cervical cancer (31). However, Simone Punt et al. (32, 33) found that increased Th17 cell infiltration was an independent protective factor associated with improved prognosis, by using immunohistochemistry and immunofluorescence staining, they found that in CSCC, IL-17 was predominantly secreted by neutrophils (66%), while Th17 cells accounted for only 4%. Such discrepancy may be due to that the function of Th17 cells should not be simply equated with the role of IL-17 during the development of cervical cancer. The tumor-promoting effects mediated by IL-17 may not originate from Th17 cells.

**Figure 1:**
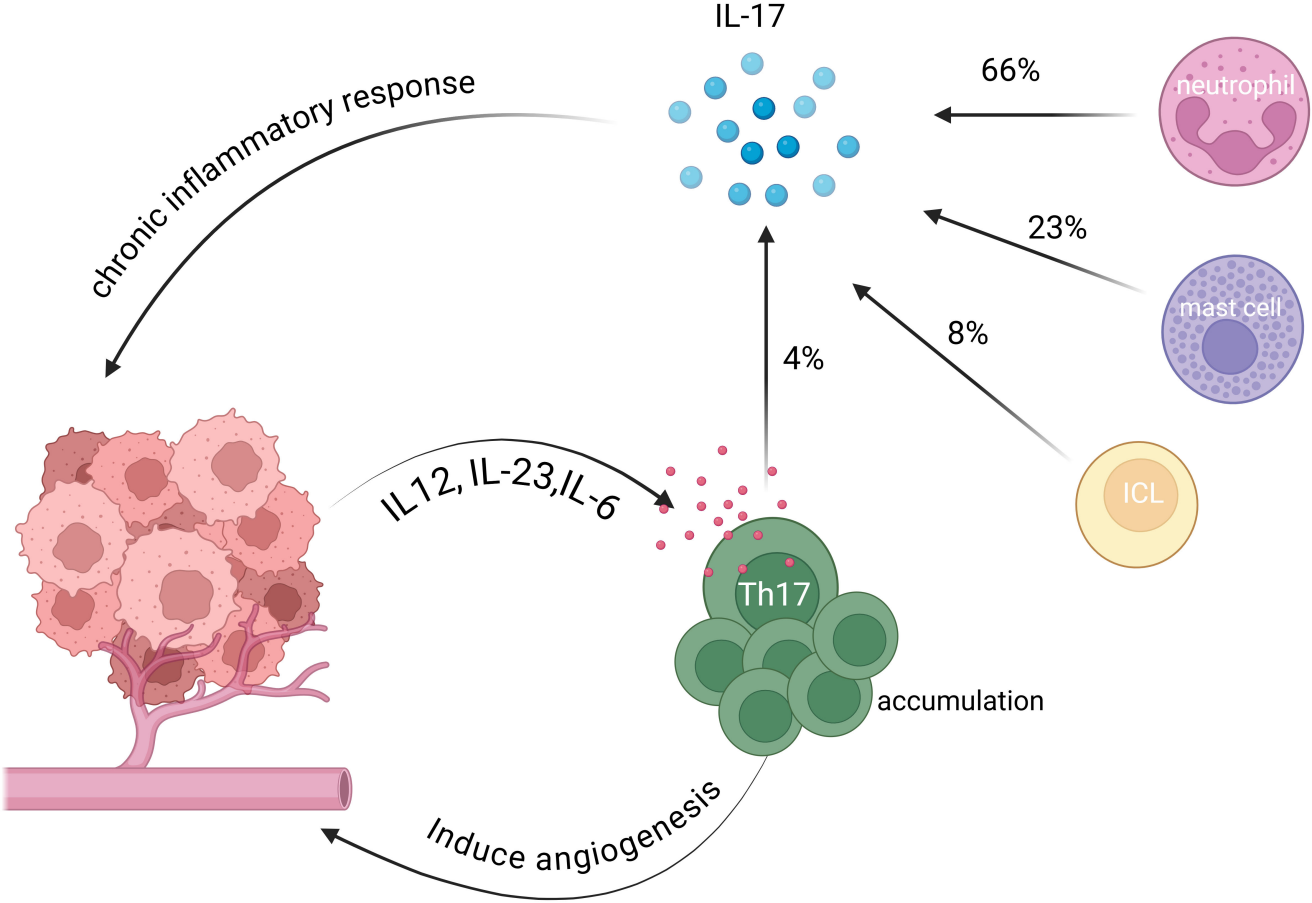
Interactions between Th17 cells and cervical cancer cells in the TME. Th17 cells are recruited to the tumor microenvironment by chemokines, including IL-12, IL-23 and IL-6, secreted by cervical cancer cells (27, 29). These cells promote tumor growth by inducing angiogenesis. Th17 cells also contribute to chronic inflammation within the tumor microenvironment by secreting IL-17, which induces an immunosuppressive state (31). Notably, some studies verified that Th17 cells account for only 4% of IL-17 secretion, whereas neutrophils, mast cells, and innate lymphoid cells contribute 66%, 23%, and 8%, respectively (33).

Thus, the functional role of Th17 cells in cervical cancer remains controversial. **Figure 1** illustrates the interactions between Th17 cells and cervical cancer cells within the tumor microenvironment through various chemokines. Future studies should move beyond the assumption that IL-17 activity directly reflects Th17 cell function and instead focus on the interactions of Th17 cells with other immune populations in the tumor microenvironment such as Treg and CD8^+^ T cells as well as their dynamic balance and functional plasticity.

#### 2.2.2 Tregs

Treg cells play a pivotal role in mediating immunosuppression within the TME, attenuating specific antitumor immune responses and enabling tumor cells to evade immune surveillance (34). The proportion of Treg cells is significantly elevated in the peripheral blood, TME, and lymph nodes of cervical cancer patients, and their high infiltration is closely associated with poor prognosis (35). Moreover, in precancerous cervical lesions, Treg cell levels are negatively correlated with spontaneous lesion regression, regardless of HPV subtype (36). These findings suggest that Treg cells play a critical immunoregulatory role in the development and progression of cervical cancer and may serve as potential therapeutic targets or prognostic biomarkers.

Various signals regulate the generation and differentiation of Treg cells in cervical cancer, thereby influencing disease progression and offering insights into immunotherapy strategies. FOXP3 is a critical marker of Treg development and function (37), it serves as the master regulator and specific molecular marker for inducible Tregs, which differentiate from naïve T cells in the periphery, and also governs the development of natural Tregs in the thymus (38). Foxp3^+^ T cells inhibit the proliferation and activation of CD8^+^ T cells (39), and their numbers increase with the progression of cervical tumor lesions (40). Neoadjuvant chemotherapy has been shown to improve the immune microenvironment of cervical cancer by reducing the number of Foxp3^+^ T cells, thereby mitigating immunosuppressive effects and enhancing antitumor immune responses (41). Endogenous STING signaling in T cells promotes the differentiation of CD4^+^ naïve T cells into Tregs and enhances FOXP3 transcription, thereby supporting Treg generation (21).FOXP1 plays an indispensable role in Tregs by facilitating FOXP3-mediated gene expression (42). NAT10 has been shown to promote FOXP1 ac4C RNA modification, leading to lactate accumulation in the TME. This accumulation enhances the immunosuppressive function of Tregs and increases PD-1 expression, thereby weakening the efficacy of PD-1 blockade therapy (43). Epigenetic modifications also contribute to Treg-mediated immune regulation, the expression levels of m6A regulatory factors are significantly associated with Tregs and plasma cells infiltration in the TME of cervical cancer (44). Notably, HMGB1 has been shown in multiple studies to modulate the immune landscape of cervical cancer by influencing the abundance and function of Treg cells. Blocking HMGB1 not only reduces immunosuppressive Tregs but also activates effector CD8^+^ T cells, thereby enhancing the efficacy of immunotherapy (45).

Highly activated human leukocyte antigen-DR regulatory T cells (HLADRhi Tregs) are the subset of Treg cells that express high levels of inhibitory molecules (46). Yang et al. found that HLADRhi Tregs were significantly more frequent in the blood of CSCC patients than in precancerous patients and healthy donors, whereas other Treg subsets showed no such difference. This increased frequency in CSCC patients contributed to a highly immunosuppressive TME, where elevated stromal HLADRhi Tregs levels were associated with reduced progression-free survival (PFS), serving as a negative prognostic indicator (47).Like CD8^+^ T cells, Treg cells also face the challenge of immune exhaustion, as they express exhaustion-related genes such as *ENTPD1* and *TOX*. However, despite their exhaustion, Tregs play a critical role in regulating T cell differentiation and activation through upregulation of genes such as *TNFRSF18 and BATF (* 15). Overall, Treg cells exhibit a clear immunosuppressive function in the cervical cancer TME, promoting tumor progression. Future research should clarify the signaling networks and epigenetic mechanisms, such as m6A modification, that regulate Treg cell function and shape the immune microenvironment in cervical cancer, to identify novel targets for immunotherapy.

### 2.3 γδT cells

γδT cells are a type of innate immune T cell with cytotoxic activity that are not restricted by MHC, accounting for only 1%–5% of peripheral blood T cells. Classified as CD4- CD8- T cells, they have attracted increasing attention in antitumor research in recent years.

Persistent HPV infection is a major etiological factor in the development of cervical cancer. γδT cells are widely distributed in the epithelial layers of the female reproductive tract, where they function alongside keratinocytes and dendritic cells as components of the mucosal immune sentinel system. Recent studies have reported significant alterations in the distribution of γδT cells in HPV-positive cervical cancer patients (48). Van Hede et al. (49) demonstrated that the oncogenic proteins encoded by HPV16 alter distribution of γδT cell in the skin of mice, with a marked increase in proangiogenic IL-17A^+^ γδT cells. In human, similar proangiogenic changes were observed specifically at the invasive squamous cell carcinoma stage, but not in precancerous lesions (50). The data suggest functional or distributional dysregulation of γδT cells may impair mucosal immune surveillance and promoting the progression of cervical cancer. Future studies should focus on elucidating how HPV modulates the recruitment, activation, and function of γδT cells. Such insights could provide a theoretical basis for the development of γδT cell-targeted immunotherapeutic strategies aimed at facilitating early HPV clearance, preventing precancerous lesion progression, and ultimately reducing the risk of cervical cancer.

γδT cells exhibit both antitumor and protumor functions in tumor immunity, exerting their effects through cytotoxicity, cell lysis, and immunomodulatory mechanisms. In various solid tumors, γδT cells have been reported to play immunosuppressive roles (51). In contrast, most current studies suggest that γδT cells predominantly exhibit antitumor activity in cervical cancer. High γδT cell infiltration is significantly associated with better prognosis (52) and a greater likelihood of benefiting from immunotherapies such as immune checkpoint inhibitors (ICIs) and tumor-infiltrating lymphocyte (TIL) therapy. This highlights a distinction between the roles of γδT cells in cervical cancer and those in other solid tumors. Such differences may be attributed to the unique viral etiology of cervical cancer. Unlike non-viral solid tumors, cervical cancer is primarily driven by HPV infection, which initially triggers a mucosal immune response and creates an early inflammatory microenvironment. As key components of mucosal immunity, γδT cells are more likely to exert their immune-activating functions and maintain cytotoxic or helper functions under these conditions. Moreover, cervical cancer, as a virus-associated malignancy, may present a greater abundance of viral antigens or stress-induced molecules (53). These ligands can be specifically recognized by γδT cells, thereby enhancing their activation and effector functions, an antigen-dependent mechanism that may be less pronounced in other types of solid tumors. Further research is needed to validate the above analysis. The antitumor effects of γδT cells in the cervical cancer TME are likely mediated primarily by direct cytotoxicity, with potential regulatory influences on the function of CD8^+^ αβT cells. It has been observed that significant signaling interactions exist between γδT cells and CD8^+^ αβT cells, particularly through the CCL5-CCR1/5 ligand-receptor axis (54). Additionally, Wang et al. identified eight γδT cell-related hub genes are upregulated in high-risk patients and are associated with poorer survival outcomes in cervical cancer (48). Interestingly, studies have shown that the correlation between γδT cells and survival weakens in patients receiving radiotherapy alone, while the survival advantage associated with γδT cells is completely lost in patients undergoing chemoradiotherapy. This suggests that γδT cell infiltration levels are not significantly correlated with survival under these treatment conditions (54). Research on γδT cells in cervical cancer is still limited, and their subset functions, plasticity, and interactions within the TME remain to be clarified.

## 3 The balance among different T cell subsets in cervical cancer

In various types of cancer patients, imbalances in T lymphocyte subset proportions are frequently observed, and cervical cancer is no exception. This imbalance can have a significant impact on the progression and prognosis of cervical cancer.

### 3.1 CD4^+^/CD8^+^

CD4^+^ T and CD8^+^ T cell expression is often suppressed or even absent in CIN and cervical cancer (55). Changes in their ratio can significantly influence the state of the immune microenvironment. A decreased CD4^+^/CD8^+^ ratio is commonly associated with poor prognosis in cervical cancer, with studies showing that patients with CSCC who have a low CD4^+^/CD8^+^ ratio exhibit worse five-year survival rates (56–58), higher lymph node metastasis rates and faster tumor growth (59, 60). However, the CD4^+^/CD8^+^ ratio in TILs is inverted in tumor tissues compared to peripheral blood, CD4^+^ cells are significantly reduced (*p* < 0.0013), while CD8^+^ cell numbers remain comparable to those in peripheral blood (*p* = 0.92), resulting in a significantly decreased CD4^+^/CD8^+^ ratio in tumor tissues, which was particularly pronounced in advanced stages of the disease (58). Meanwhile, studies have found that patients with a lower Treg/CD8^+^ T cell ratio in primary tumor tissues exhibit prolonged survival, especially when accompanied by abundant M1 macrophages, further improving survival (61, 62). Neoadjuvant chemotherapy (NACT) can also influence the balance of immune cells in the TME of cervical cancer patients. In post-NACT patients, the peritumoral Foxp3^+^ T cell to intratumoral CD8^+^ T cells ratio serves as a critical predictor of survival, with lower ratios being positively correlated with improved PFS and overall survival in locally advanced cervical cancer (41). Therefore, adjusting the CD4^+^/CD8^+^ ratio, in combination with therapeutic interventions such as radiotherapy or chemotherapy may significantly improve patient survival outcomes (63).

Collectively, the balance among different T cell subsets significantly influences the progression and prognosis of cervical cancer. This suggests that T cell subsets do not function in isolation within the immune microenvironment but interact extensively through mechanisms that remain to be elucidated.

### 3.2 Th/Treg

In normal cervical tissues, balance between Th cells and Tregs is maintained, reflecting the immune system’s capacity to combat tumors while preventing excessive immune responses. However, this balance is disrupted in cervical lesions (64). In high-grade squamous intraepithelial lesions (HSIL), the number of Th cells exceeds that of Tregs, suggesting an active immune response to potential carcinogenesis. In contrast, in cervical cancer, Tregs outnumber Th cells, leading to immune suppression that facilitates tumor immune evasion (15). Interestingly, Zhang et al. reported no significant difference in the Th17/Treg ratio between CIN and cervical cancer in peripheral blood. This may be attributed to the significant differences in immune microenvironment composition between tumor tissues and the peripheral circulation. However, compared with healthy cohorts, both CIN and cervical cancer patients exhibit a significantly altered Th17/Treg ratio in peripheral blood. This finding suggests that an imbalance in the Th17/Treg cell ratio is present throughout the progression of cervical lesions in peripheral circulation. Nevertheless, this imbalance does not appear to show a marked shift from CIN to cervical cancer. The significant increase in Treg cells within tumor tissues may play a critical role in facilitating immune evasion (30). Furthermore, other studies have reported that the Th17/Treg ratio is significantly higher in cervical cancer patients with lymph node metastasis or invasion compared to cancer-free individuals (65). This imbalance may promote angiogenesis, thereby contributing to further cervical cancer progression (28).

## 4 Interaction of T cells with other immune cells in cervical cancer

In the cervical cancer TME, various immune cells engage in complex interactions with T cells, collectively shaping the immune landscape and regulating tumor initiation and progression. The immune cell populations within the TME include T and B lymphocytes, tumor-associated macrophages, dendritic cells (DCs), natural killer cells (NK cells), neutrophils and myeloid-derived suppressor cells (MDSCs). These intricate cellular interactions not only influence the functional state of T cells but also shape the immunosuppressive or antitumor properties of the cervical cancer TME to varying degrees, ultimately affecting disease progression and the efficacy of immunotherapy.

### 4.1 DCs and T cells

DCs often interact with T cells in the cervical cancer TME. Studies have shown that plasmacytoid DCs (pDCs) and Treg cells co-localize in metaplastic and (pre)neoplastic cervical lesions. Exposure to the cervical or vulvar TME enables pDCs to promote the differentiation of naïve CD4^+^ T cells into Tregs (66), further enhancing the immunosuppressive state of the tumor. Meanwhile, interactions between CD8^+^ T cells and dendritic cells have been extensively studied. In tumor-draining lymph nodes (t-LNs), the proliferation and activation of CD8^+^ T cells correlate with dendritic cells homing (67). Further studies have revealed that impaired CD8^+^ T cells recruitment in cervical adenocarcinomas (CAde) is associated with reduced levels of cDC1s (classical dendritic cells subtype 1), leading to decreased production of T cell-attracting chemokines CXCL9 and CXCL10 (68). This chemokine deficiency is considered a key factor contributing to the poor immune response and prognosis observed in CAde patients (69). Additionally, DCs have been found to influence T cell function through complex immunoregulatory mechanisms. Qu et al. demonstrated that LAMP3^+^ DCs (lysosomal-associated membrane protein 3-positive dendritic cells) suppress exhausted CD8^+^ T cells *via* immune checkpoints. Additionally, these cells secrete chemokines to attract and activate Tregs and stimulate immune checkpoint molecules, further enhancing immunosuppression in cervical cancer. This process creates a favorable environment for tumor growth and metastasis (15).

### 4.2 Myeloid cells and T cells

The interactions between myeloid cells and T cells within the cervical cancer TME have also been extensively studied. Galliverti et al. demonstrated that myeloid cells suppress CD8^+^ T cells and antigen-presenting cells in the cervical cancer TME, thereby limiting the efficacy of immune responses against cancer and resulting in poor immunotherapy outcomes (70). In a mouse model of cervical cancer, Silveira et al. demonstrated that the administration of immuno-modulatory drug Swainsonine promoted the accumulation of MDSCs, which significantly inhibited antitumor T cell responses, particularly the activity of CD8^+^ T cells (71). Moreover, higher levels of PD-L1-positive MDSCs, have been observed in cervical cancer patients, where they suppress T cell activity (72). M0 macrophages can be induced to polarize into either M1 or M2 macrophages. Chen et al. found that interactions between M0 macrophages and naïve CD4^+^ T cells may contribute to the immunosuppressive microenvironment in cervical cancer. During the transition from a normal to a tumor state, increased interactions between M0 macrophages and naïve CD4^+^ T cells were observed (73). Differentially expressed genes in M0 macrophages were significantly enriched in the Th17 cell differentiation pathway, suggesting that M0 macrophages are likely to influence the differentiation of naïve CD4^+^ T cells. In contrast, Steen Wijk et al. demonstrated that high infiltration of CD14-positive, CD33-negative and CD163-negative M1 macrophages in the tumor epithelium was associated with significantly prolonged disease-specific survival in cervical cancer patients. These M1 macrophages promote CD8^+^ T cell infiltration, highlighting the distinct roles of different macrophage subsets in modulating T cell responses (62).

**Figure 2:**
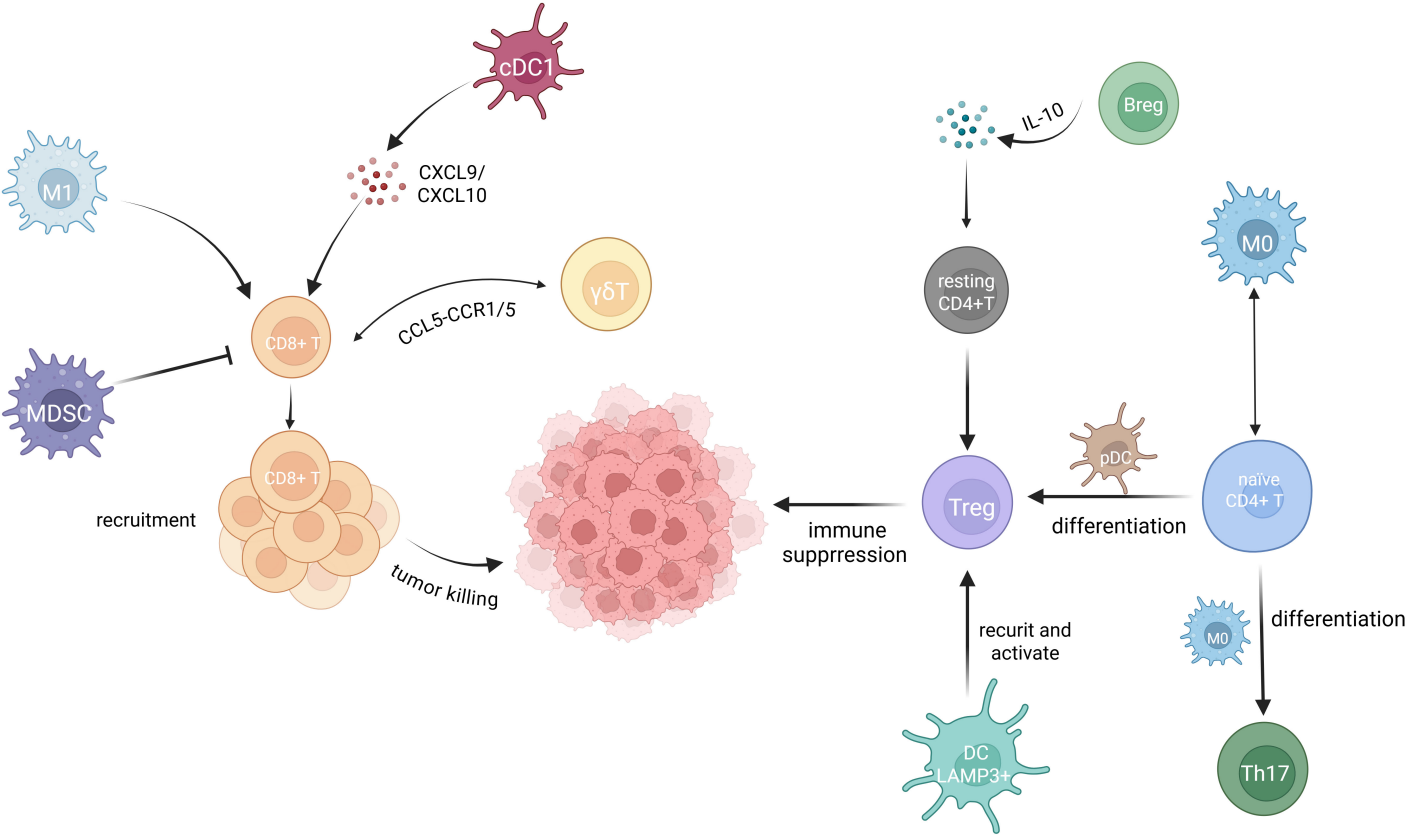
Immune cell interactions in the TME of cervical cancer. Extensive interactions among immune cells occur within the TME of cervical cancer. Treg cells are well known for their immunosuppressive role in the cervical cancer TME. Breg cells convert resting CD4^+^T cells to Treg cells through IL-10 secretion (35). LAMP3^+^ DCs recruit and activate Treg cells (15). Naive CD4^+^ T cells differentiate into Treg cells in response to pDCs (66) and into Th17 cells when influence by M0 macrophages. Enhanced interactions between M0 macrophages and naive CD4^+^ T cells are observed as tumor progress (73). M0 macrophages further differentiate into either M1 or M2 macrophages. M1 macrophage facilitate CD8^+^ T cell infiltration into the TME, and CD8^+^ T cells exhibit a well-established cytotoxic effect against cervical cancer cells (62). cDC1 cells recruit CD8^+^ T cells by secreting the chemokines CXCL9 and CXCL10 (69). MDSCs strongly suppress the activity of CD8^+^ T cells (71). γδT cells communicate with CD8^+^ T cells through the CCL5-CCR1/5 axis (54).

In addition, Sims et al. reported that CD4^+^ T cells infiltrating the cervical cancer TME can support and enhance the activity of other immune cells by releasing T cell cytokines (74). Yang et al. observed a positive correlation between the percentages of Bregs and Tregs in cervical cancer, with CD4^+^Foxp3^+^ Tregs being induced by Bregs (35). **Figure 2** summarizes the interactions among immune cells in the cervical cancer TME discussed above.

## 5 Functional variations of T cell in different pathologic types of cervical cancer

CSCC and CAde are the two main pathological subtypes of cervical cancer, but their immune microenvironments, particularly in terms of T cell infiltration and functional states, exhibit significant differences that remain incompletely understood.

There are distinct differences in T cell infiltration between CSCC and CAde. Rotman et al. reported that T cell infiltration levels were significantly higher in CSCC than in CAde, potentially due to differences in T cell migration. However, in t-LNs, CAde patients exhibit higher levels of differentiated CD8^+^ memory T cells, including central memory T cells and effector memory T cells, whereas the t-LNs of CSCC contain exhibit a higher proportion of naïve CD8^+^ T cells. This may reflect greater antigen stimulation and memory T cell formation in the immune systems of CAde patients (69).

Pathological subtype also appears to influence T cell function in cervical cancer. In CSCC, Tregs were abundant in the TME, reflecting stronger immunosuppression. However, the presence of Tregs is positively correlated with survival in CAde patients. This suggests that the role of Treg cells may vary across different pathological subtypes. Similarly, Punt et al. highlighted that IL-17^+^ cells represented a beneficial immune response in CSCC, correlating with improved survival, whereas Th17 cells in CAde were found to promote tumor progression and poor prognosis (75). Lin et al. analyzed the immune characteristics of CSCC and CAde, found that both cancer subtypes exhibit abundant CD8^+^ T cells and naïve T cells within their TME. Exhausted T cells were more predominant in CAde. whereas CD8^+^ T cells in CSCC patients exhibited a higher cytotoxicity score (76). In contrast, Rotman et al. found that the expression levels of immune checkpoints on CD8^+^ and CD4^+^ T cells were higher in CSCC patients than in CAde patients, suggesting a more pronounced exhausted T cell phenotype in CSCC patients (69).

In summary, T cells in different pathological subtypes of cervical cancer exhibit significant differences in immune infiltration levels and functional differentiation states, potentially influencing therapeutic responses and prognoses. Currently, CAde patients receive the same treatment regimens as CSCC patients yet typically exhibit poorer therapeutic outcomes (69). These findings highlight the need for precision immunotherapy tailored to the specific pathological subtypes of cervical cancer.

## 6 Differences in T cell subset infiltration patterns from precancerous lesions to cervical cancer

The infiltration patterns of T cells undergo continuous changes from the precancerous stage to later stages, reflecting the progressive dysregulation of the TME and key features of immune system regulation during tumor progression. Compared to the precancerous stage, the ratio of CD4^+^/CD8^+^ T cells in the TME of cervical cancer patients is significantly increased, with CD4^+^ T cells gradually becoming more predominant in the immune microenvironment. Among them, activated CD4^+^ T cells increase progressively, while resting CD4^+^ T cells decrease. Additionally, Tregs also show a gradual increase with the severity of cervical lesions (66, 77). However, despite the declining proportion of CD8^+^ T cells, no significant changes are observed in the numbers of resting and activated cells. Notably, CD8^+^ T cells exhibit a distinct pattern, with their numbers being lowest in normal tissues and progressively increasing with disease severity, reaching a peak at the HSIL stage (78). High levels of tissue-resident CD8^+^ T cell infiltration (24) have been observed in HSIL tissues, whereas tumor tissues show substantial enrichment of exhausted CD8^+^ T cells (79). These findings suggests that immune activation and suppression occur simultaneously during the precancerous stages of cancer development, supporting the concept of immune surveillance at early stages, while HSIL represents a critical balance point between activation and suppression.

For patients already diagnosed with cervical cancer, compared to stage I cervical cancer patients, those in stage II exhibit stronger immunosuppressive features, including decreased levels of circulating Th1 cells and elevated levels of Th2 cells, Th17 cells, and Tregs (80). These changes further exacerbate the immunosuppressive microenvironment, which is characterized by processes such as dendritic cell immaturity, the differentiation of CD4^+^ T cells into Tregs, and the polarization of immunosuppressive macrophages (36).

In summary, as cervical lesions progress, the TME exhibits increasingly pronounced immunosuppression and T cell exhaustion. While this trend partly reflects the irreversible nature of advanced disease, it also suggests that effectively reversing CD8^+^ T cell exhaustion may offer greater therapeutic benefits for patients in later stages.

## 7 Dynamic changes of T cells in cervical cancer immunotherapy

In recent years, immunotherapy has made significant strides, markedly extending the survival of patients with advanced or metastatic cancers that were previously considered incurable (81), and T cells play a pivotal role in the cellular immunotherapy of cervical cancer. However, due to inter-individual variability, only a small subset of cervical cancer patients is likely to be benefit from immunotherapy (3). As such, elucidating the mechanisms by which T cells influence immunotherapy efficacy is essential for improving patient outcomes.

Immune checkpoint inhibitors (ICIs) have gained increasing attention in cervical cancer treatment due to their potential to restore T cell-mediated antitumor immunity, among which CTLA-4 and PD-1/PD-L1 are the most extensively studied regulators. Tumor cells exploit these negative regulatory mechanisms to suppress immune responses and evade immune surveillance (82). As one of the most well-characterized immune checkpoint pathways, PD-1/PD-L1 has emerged as a critical therapeutic target in cervical cancer immunotherapy. Its blockade can promote T cell infiltration into the tumor microenvironment and enhance antitumor activity. Studies have shown that PD-L1 expression is positively correlated with the density of CD8^+^ TILs and PFS, particularly in cervical cancers with destructive invasion patterns (83). PD-L1 inhibitor durvalumab local injection substantially alters the immune microenvironment of cervical cancer and their draining lymph nodes, including reducing the presence of Tregs and PD-L1-positive macrophages in tumors, thereby enhancing T cell-mediated antitumor activity (84). CMTM6 has been identified as a key regulatory factor of PD-L1 in CAde and plays a crucial role in the regulation of T cell exhaustion (85, 86). This is supported by the findings that cervical cancer patients who respond favorably to immunotherapy typically exhibit higher levels of CMTM6 and PD-L1 expression. Liang et al. further demonstrated that high CMTM6 expression is closely associated with higher infiltration rates of CD8^+^ T cells in HPV-associated adenocarcinoma (HPVA), suggesting that CMTM6 is an important contributor to shaping the immune landscape of HPVA (87). ICIs have been widely applied in cancer treatment, however, the efficacy of ICI in cervical cancer remains relatively low, partly due to the accumulation of MDSCs in the TME, which suppress T cell function. In this context, Liang et al. demonstrated that all-trans retinoic acid inhibits MDSCs function, thereby enhancing the efficacy of anti-PD-L1 therapy in cervical cancer (72). In addition to immunosuppressive cells, the composition and extent of immune cell infiltration within the TME may significantly influence both the selection and effectiveness of immunotherapy. Chen et al. proposed that profiling immune infiltration could assist in tailoring immunotherapy strategies for cervical cancer patients. Their findings revealed that patients with high ICI scores but low CD8^+^ T cell infiltration were more likely to benefit from anti-PD-L1 therapy, while those with lower ICI scores might respond better to anti-CTLA-4 treatment (88). These results underscore the importance of accurate TME characterization, particularly T cell-related features, in guiding immunotherapeutic decisions.

In addition to the well-established CTLA-4 and PD-1 pathways, a growing number of other immune checkpoints involved in T cell regulation are being investigated as new directions for immunotherapy development. B7-H4, a member of the B7 immune checkpoint molecule family, suppresses antitumor immune responses within the cervical tumor microenvironment, and its high expression is significantly associated with poor prognosis in cervical cancer patients (89). B7-H4 inhibits the infiltration and immune function of CD8^+^ T cells, corresponding to the reduction of IFN-γ production, while having no significant effect on the infiltration of CD4^+^ T cells or Foxp3^+^ Tregs. As a potential therapeutic target for cervical cancer immunotherapy, B7-H4 blockade therapies may help restore T cell function and enhance antitumor immune responses (90).

Given the central involvement of T cells in antitumor immunity, factors that influence PD-1/PD-L1 expression can shape the immune microenvironment by altering T cell quantity and function, thereby affecting the immunotherapeutic response in cervical cancer. Recent studies have shown that latent Epstein–Barr virus (EBV) infection can exacerbate immune evasion by increasing the number of Tregs and CTLA-4 tumor-infiltrating lymphocytes (TILs), as well as upregulating PD-1/PD-L1 expression. These changes collectively compromise CD8^+^ T cell activity, thereby facilitating tumor progression. Consequently, EBV-infected patients may represent promising candidates for ICI-based therapies (91). Notably, conventional cancer treatments such as radiotherapy and chemotherapy also impact immune checkpoint expression and T cell status. Herter et al. reported that following chemoradiotherapy, the frequency of inhibitory receptors, including PD-1 and TIGIT, on tumor-infiltrating CD8^+^ T cells was markedly reduced, while PD-L1 expression on circulating CD8^+^ T cells was increased (92). Similarly, concurrent CCRT in cervical cancer has been shown to suppress the immune microenvironment in both peripheral blood and tumor tissue by reducing T cell counts, T cell receptor diversity, and PD-1/PD-L1 expression. These findings suggest that administering ICIs before initiating CCRT may be more effective than during or after the treatment (93, 94). Taken together, these findings emphasize the importance of incorporating T cell status within the tumor microenvironment into the design and optimization of PD-1/PD-L1-based immunotherapeutic strategies for cervical cancer. **Table 2** provides a detailed summary of the factors influencing cervical cancer prognosis mentioned in the text.

**Table 2:** Summary of factors influencing prognosis in cervical cancer.

Category	Specific Factor	Effect Direction	Mechanism or Observation	Contradictory Findings
Balance Among T Cell Subsets	Th17/Treg	Positive	Significantly elevated in patients with lymph node metastasis and invasion in cervical cancer (28, 65).	Zhang et al. reported no significant difference in this ratio between CIN and cervical cancer in peripheral blood (30).
	CD4^+^/CD8^+^	Positive	In the cervical cancer TME, CD4^+^ T cells are markedly reduced (61), while CD8^+^ T cell levels remain unchanged (58).	N/A
	CD8^+^/Treg	Positive	Higher ratios are associated with prolonged survival (61), particularly in the presence of M1 macrophages (62).	N/A
Cytokines	IL-33	Positive	Promotes antitumor activity of CD8^+^ T cells (16).	IL-33 stimulates ILC2s to secrete IL-13, which modulates M-MDSCs, enhancing their immune evasion capacity (17)
Molecular Markers	GLUT1	Negative	High GLUT1 expression weakens CD8^+^ T and Th1 cell activity, facilitating immune evasion (18).	N/A
	BATF2	Positive	BATF2 deficiency leads to CD8^+^ T cell exhaustion and reduced infiltration (20).	N/A
	STING	Negative	T cell-intrinsic STING expression negatively correlated with CD8^+^ T cell density; promotes CD4^+^ naïve T cell differentiation into Tregs (21).	Higher expression of downstream STING pathway genes (e.g., *CCL5, CXCL10*) is associated with better survival in cervical cancer patients (22).
	NAT10	Negative	Promotes lactate accumulation in the TME, enhancing Treg immunosuppressive function and increasing PD-1 expression (43).	N/A
Immune Checkpoints	B7-H4	Negative	Inhibits CD8^+^ T cell immune functions and reduces IFN-γ production (90).	N/A

N/A indicates no contradictory findings were identified in this reviewed literature.

E6 and E7, the key oncogenic proteins of HPV, are critically involved in sustaining proliferative signaling, evading tumor suppressors, activating telomerase and inducing angiogenesis and metastasis. As a result, vaccines targeting E6 and E7 have emerged as a major focus in cervical cancer treatment research, as they can enhance T cell infiltration within the tumor, reverse the immunosuppressive microenvironment and ultimately achieve favorable therapeutic outcomes. Zhen et al. developed a liposome-based delivery system using CRISPR/Cas9, which effectively knocked out HPV E6/E7 genes, triggering the release of damage-associated molecular patterns, including HMGB1 and ATP, and activating antitumor immune responses. This system also significantly enhanced CD8^+^ T cell infiltration while reducing Treg cells and increasing the expression of pro-inflammatory cytokines, such as IL-12, IFN-γ and TNF, effectively reversing the immunosuppressive tumor microenvironment. Moreover, the approach demonstrated enhanced antitumor effects when combined with anti-PD-1 therapy, significantly reducing tumor volume and prolonging progression-free survival in mice (95). Wang et al. synthesized a bacterial vaccine, Salmonella-9RE7, an arginine-extended HPV E7 antigen, which effectively induced E7-specific CD8^+^ T cell immunity, significantly inhibiting tumor growth and improving survival rates in mice. Additionally, combining this treatment with Alb-IFNβ increased CD8^+^ and CD4^+^ T cell infiltration in the tumor microenvironment while reducing PD-1 expression on TILs and regulatory T cells, thereby enhancing the overall immune response (96). The vaccine PRGN-2009 targets the viral oncogenic proteins E6 and E7, significantly increasing the number of CD8^+^ and CD4^+^ T cells within the tumor microenvironment. These T cells exhibit functional diversity, secreting IFN-γ and granzyme B, which are critical for effective cytotoxic responses. Studies suggest that combining PRGN-2009 with immune checkpoint inhibitors, such as anti-PD-1 or anti-PD-L1, may further enhance therapeutic efficacy (97). Currently, the combined delivery strategy of E6/E7-targeted vaccines with chemotherapeutic agents like cisplatin is emerging as a new research focus. Meanwhile, all the above studies were conducted in animal models, and their safety remains to clarified before clinical translation can be achieved. Further improving the tumor-targeting ability of the vaccine is expected to enhance its efficacy while ensuring treatment safety, thereby contributing to a more optimal clinical application.

An increasing number of studies have shown that, compared to monotherapy, the combination of immune checkpoint inhibitors, mRNA vaccines, and chemotherapeutic agents yields superior clinical outcomes. These immunotherapeutic approaches act *via* distinct mechanisms, and their combination offers complementary effects. Therapeutic vaccines can induce antigen-specific T cell responses; however, the functionality of these T cells is often constrained by immunosuppressive factors within the TME. Rice et al. reported that the HPV vaccine Ad5 [E1-, E2b-]-E6/E7 enhances the infiltration of CD8^+^ T cells in tumor, yet these T cells remain functionally impaired. Co-administration of PD-1 antibodies effectively blocks the PD-1/PD-L1 axis, reverses T cell exhaustion, and significantly augments the vaccine-induced antitumor response (98). Similarly, Sluis et al. demonstrated that while synthetic long peptide (SLP) vaccines elicit antigen-specific CD8^+^ T cell responses, early and excessive expansion may lead to T cell dysfunction or regulatory suppression. Topotecan a topoisomerase I inhibitor, can transiently suppress dendritic cell activity and delay CD8^+^ T cell expansion. When combined with vaccination, this strategy avoids early overstimulation and exhaustion, promotes sustained T cell proliferation and effector function, and ultimately prolongs survival (99). Collectively, the favorable efficacy of combination immunotherapy can be largely attributed to the synergistic effects of different therapeutic strategies targeting multiple key steps of the immune response, including antigen presentation, T cell activation, and the reversal of immunosuppression. Thus, weakening immunosuppressive mechanisms and enhancing immune-activating signals, thereby remodeling the tumor microenvironment is also one of the common strategies for combination therapy. The E7-TriMix mRNA vaccine effectively induced HPV-specific CD8^+^ T cells, demonstrating significant antitumor effects, particularly in non-mucosal tumor sites. When combined with cisplatin, it significantly reduced the levels of MDSCs and Tregs in the tumor microenvironment, further enhancing the antitumor activity of CD8^+^ T cells (100). However, current findings on combination drug therapies are primarily derived from mouse models. Although several preclinical studies have demonstrated the superior efficacy of combination immunotherapy, its clinical translation remains challenging. For instance, while topotecan has been shown to enhance T cell function, it can also cause myelosuppression and neutropenia, thereby limiting its dosage in human patients. Similarly, although the Ad5 vector vaccine is highly immunogenic, its viral vector is easily recognized and eliminated by the host immune system, potentially triggering anti-vector immune responses that may diminish the efficacy of subsequent vaccinations. Moreover, patients’ responses to vaccines are heterogeneous, and identifying the populations most likely to benefit from such therapies remains an unresolved issue. Therefore, caution is warranted when extrapolating these preclinical results to human clinical applications.

T cells are the central effector cells in cervical cancer immunotherapy, and their functional status directly influence treatment strategies and patient prognosis. With the ongoing advancement of immune checkpoint inhibitors, therapeutic vaccines, and combination regimens, modulating the TME to enhance T cell activity has emerged as a major research focus. In recent years, the development of immunohistochemistry and high-throughput multi-omics technologies has facilitated a shift toward personalized immunotherapy. Patients with high PD-L1 expression, enriched TILs (101), or an elevated Treg/CD8^+^ T cell ratio may be more responsive to combination therapy involving PD-1 blockade. For immunologically “cold” tumors, strategies aimed at reprogramming the TME, such as the use of STING agonists (102), low-dose chemotherapy, or vascular-disrupting agents followed by vaccination may help to maximize immune activation. However, these approaches require further experimental validation. Future studies should aim to identify reliable predictive biomarkers, optimize dosing and scheduling strategies, and develop translational combination therapies to improve response rates and achieve durable clinical benefits in patients with cervical cancer.

## 8 Conclusion

Cervical cancer is one of the most common malignant tumors in women, with different T cells subsets playing a central role in cellular immunity and performing critical functions within the TME. CD8^+^ T cells play a central antitumor role in cervical cancer, but their function is often limited by immune exhaustion within the TME and this phenomenon becomes more pronounced in advanced stages of the disease. Therefore, reversing CD8^+^ T cell exhaustion is considered a key strategy to enhance the efficacy of immunotherapy and delay disease progression. In contrast, CD4^+^ T cell in cervical cancer are generally associated with immunosuppressive functions, though their roles remain controversial. The immunosuppressive activity of Treg cells has been widely reported, but new evidence suggest that their function may vary depending on histological subtype, and that Tregs themselves may also exhibit exhaustion phenotypes similar to those of CD8^+^ T cells. These findings highlight the need for further investigation into the regulatory mechanisms governing Treg differentiation during disease progression and the dynamic changes in their immunosuppressive capacity. Research on Th17 cells has primarily focused on their secretion of IL-17; however, recent findings indicate that IL-17 may originate from various cells other than Th17. Therefore, future research should move beyond the conventional assumption that IL-17 activity directly reflects Th17 function, and instead focus on exploring the immunoregulatory roles of Th17 cells themselves, as well as their interactions with other immune or stromal components within the TME. Unlike in other solid tumors, γδT cells serve as a key component of the mucosal immune barrier and exhibit clear antitumor activity in cervical cancer. This may be attributed to the unique virus-related etiology of the disease. Future studies should clarify the specific role of γδT cells in HPV clearance and their interactions with other immune cells, provide new insights for the development of targeted immunotherapeutic strategies.

The interactions among various T cell subsets, along with their complex crosstalk with other immune cells, underscore the delicate balance between immune activation and suppression within the cervical cancer TME and highlight key mechanisms of tumor immune evasion. Investigating the infiltration patterns and functional dynamics of T cell subsets across different stages of cervical cancer and pathological subtypes is essential for understanding disease progression and advancing the development of novel immunotherapeutic strategies. With the ongoing advancement of immunotherapy, achieving more precise and personalized treatment has become a major research focus. Accordingly, systematic characterization of the cervical cancer immune microenvironment across different pathological types and disease stages, and tailoring immunotherapeutic strategies based on these distinct immune profiles, is expected to further improve clinical outcomes. Such efforts are expected to enhance therapeutic efficacy, improve patient prognosis, and advance the field of cervical cancer immunotherapy.
